# Patients’ bill of rights and effective factors of workplace violence against female nurses on duty at Ilam teaching hospitals

**DOI:** 10.5249/jivr.v9i1.779

**Published:** 2017-01

**Authors:** Ali Ashraf Aivazi, Waleyeh Menati, Hamed Tavan, Sasan Navkhasi, Abuzar Mehrdadi

**Affiliations:** ^*a*^Department of Public Health, Ilam University of Medical Sciences, Ilam, Iran.; ^*b*^Department of Nursing, Psychosocial Injuries Research Center, Ilam University of Medical Sciences, Ilam, Iran.

**Keywords:** Hospitals, Iran, Nurses, Patients' rights, Workplace violence

## Abstract

**Background:**

Workplace violence against female nurses is an increasing problem. In addition, recognizing the rights of patients can reduce such violence against female nurses. Therefore, the aim of the current study was to investigate workplace violence against female nurses in respect of patients' bill of rights at two public hospitals in Ilam in 2012.

**Methods:**

In a descriptive cross-sectional research, workplace violence against female nurses was studied. Data were gathered employing a researcher made questionnaire filled out by 106 female nurses. The questionnaire was on workplace, physical and verbal violence of patients and their attendants, and also the patients' rights as respected by nursing staff. Permission of university ethics committee was obtained. Data analyses were done by T-test and ANOVA in SPSS software.

**Results:**

Totally, 90.6 % and 15.1 % of the participants were subjected to verbal and physical assaults by patients, respectively during last year of the study. Further, 92.5% and 11.3% of nurses experienced verbal and physical assaults by the patients' attendants, respectively. Most of the nursing staff believed that reporting aggressive attacks to the concerned officials would not be useful. A negative significant correlation was found between the aggressions of patients with age as well as marital status of nurses, (P= 0.04). Furthermore, a significant association was seen between physical violence of patients and the nurses’ recognition of the patients' bill of rights (P= 0.03).

**Conclusions:**

Due to high rate of violence against female nurses, some proper and effective actions such as employing a trained security force along with legal punitive charges against violators through responsible officials are highly suggested.

## Introduction

Literature shows that societies are becoming more violent, and the degree of aggressiveness at workplace is globally increasing as well.^1,2^ Workplace violence is a disturbing problem for many health care practitioners around the world, ^3^ which includes intimidation, verbal or physical threats, physical attack, property damage, and even sexual harassment. ^4-6^ Administrations’ reports demonstrate that nurses are at higher risk (about three times) of workplace assaults than other professions. ^7-11^ By workplace violence, health care delivering process is degraded and leads to a hostile environment which jeopardizes the goal of providing proper care. ^12-15^ Unsatisfied staff will enhance indirect costs of health system through job turnover, decreased productivity, absenteeism, and decreased productivity of health team, which all can be related to unresolved issues of workplace violence. ^16-19^ A hospital-based health care system should obey the rights of patients and their families, ^20^ which in turn can result in the reduction of workplace violence against staff. 

Unfortunately, the prevalence of workplace violence against nurses is relatively high, and some researchers have reported prevalence of 62 % and even up to 95%^21-22^ but the reports of workplace violence seem to be almost inaccurate. ^23^ The present study aimed at investigating the workplace violence and its contributors in two teaching hospitals in Ilam, Iran, in 2012. 

## Methods

In a cross-sectional study, out of 250 registered nurses at different wards, a sample of 106 people in two public hospitals in Ilam namely Imam Khomeini and Shahid Mostafa Khomeini were surveyed. 

The study design and instruments were approved by ethics committee of the hospitals prior to the study. The study was conducted from July to August 2012, and the data were collected by a questionnaire on workplace violence during the last year of the study. 

The questionnaire contained 26 items consisting of demographic data (age, job history, marital status, academic degree and the working ward) along with some phrases on verbal/ physical violence of patients, verbal/ physical violence of patients' visitors (5 items), and nursing reactions while experiencing violent events (9 items). 

Further, the recognition of patients’ bill of rights was rated based on a Likert-score (0 for never and 6 for always) by 21 items in the questionnaire. For the bill of rights part, the minimum and maximum scores were zero and 126 respectively, so that the scores less than 47.25, 47.26-73.5, 73.6-99.76, and finally more than 99.76 scores were considered low, medium, good and excellent, respectively. 

The questionnaire on the Patient’s Bill of Rights contained 21 items in four main themes: a) right of receiving health services in a respectful manner and free from any discrimination, 3 items; b) right of access to information, 10 items; a) right of autonomy and decision-taking, 4 items; and right to submit complaints on medical errors, 4 items.^11^

The validity and reliability of the questionnaires were calculated using the CVI and test-retest methods. The reliability and consistency analysis of the questionnaire performed using the Cronbach's coefficient, which showed α of 0.78 for the whole sample. Data analysis was conducted using two-independent-samples' T-test, and one-way analysis of variance (ANOVA) for comparing more than two groups, in SPSS ver. 19. Table 1 indicates demographic data of the studied nurses. Normality of the data was checked through the Kolmogorov-Smirnov test. The statistical significant difference/ association of the variables was set at P<0.05.

**Table 1:** Characteristics of study sample

Demographic information		No. (%)
Academic Degree	Bachelor Vocational	86(81.3) 20(18.7)
Age	<30 years 30-40 years >40 years	43(40.6) 48(45.3) 15(14.1)
Marital status	Single Married	20(18.7) 86(81.3)
Shift type	Fixed Circulating	2(1.9) 104(98.1)
Job experience	<5 years 5-9 years 10-14 years ≥15 years	27(25.4) 29(27.4) 6(5.7) 44(41.5)
No. Shifts per week	<7 shifts 7 shifts >7 shifts	19(18) 23(19.8) 64(62.2)

## Results

**Figure 1:**
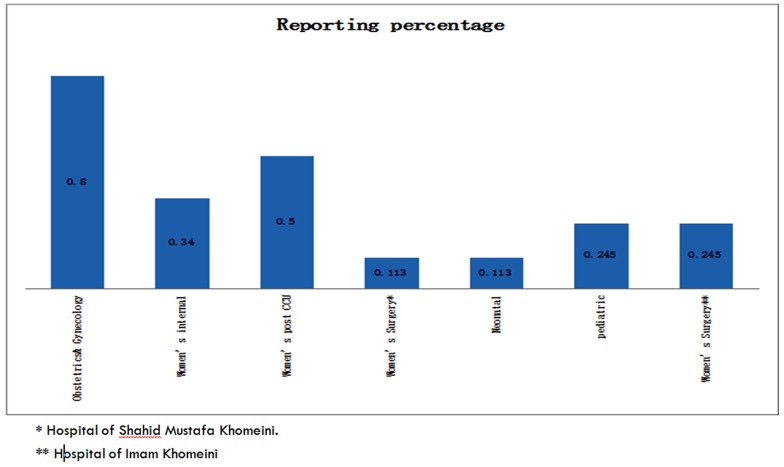
The rate of reported cases of violence in different wards of the teaching hospitals during the year prior to the study.

Out of all the studied nurses, 90.6% had experienced verbal violence from patients, 15.1 % physical violence and 92.5 % of them had been assaulted verbally by the patients’ attendants while 11.3% had been attacked physically. Table 2 shows exposure to violence by nurses at work during the past year. Table 3 displays the nurses' exposure to the patients and their attendants' violent attacks during last year. Figure 1 displays reported cases of violence in different wards of both hospitals in the year prior to the investigation.

**Table 2:** Exposure of female nurses to violence at work during last year.

Workplace ward	Violence type					
	Patient companion's threat with weapon (%)	Patient threats with weapon (%)	Patient companion's physical assault (%)	Patient companion’s verbal assault (%)	Patient's physical violence (%)	Patient's verbal assault (%)
Obstetrics & Gynecology	20	0	0	100	80	100
Women Internal	0	0	0	100	0	100
Women Post CCU	15	15	15	85	15	85
Women Surgery*	17	0	0	100	0	100
Neonatal	0	0	34	100	0	67
Pediatrics	16	16	16	84	8	92
Women Surgery**	31	23	8	92	16	92

* Shahid Mostafa Khomeini Hospital ** Imam Khomeini Hospital Note: In the current table, more than one option was selected by respondents; hereby the column summation is more than 100%.

**Table 3:** Nurses exposure to different violent attacks of patients and their companions

The number of violent attacks	Physical assault by patients' companion (%)	Verbal assault by patients' companion (%)	Physical assault by patients (%)	Verbal assault by patients (%)
Never	79.2	9.4	77.4	9.4
5>	17	34	15.1	30.2
10-5	1.9	9.4	1.9	15.1
10<	1.9	47.2	5.6	45.3

A relationship was observed between the patients' bill of rights and physical assaults by patients and their attendants. So, while the more the patients' bill of rights was observed, the less the rate of aggressive events (physical violence) was experienced by the nurses. However, no relationship was observed between the recognition of patients' bill of rights and verbal assaults (P=0.063). Furthermore, a significant relationship was found between physical violence of patients and respecting the patients' rights (P= 0.03). In Table 4, the relation between violence and patients’ bill of rights has been analyzed using ANOVA, regression and x^2^.

**Table 4:** The relationship between violence and patients’ bill of rights using ANOVA, regression and x2.

Patients’ bill of rights and Physical assault by patients' companion	ANOVA	X^2^	Regression
Patients’ bill of rights and Verbal assault by patients' companion	**0.035**	38.2	0.85
Patients’ bill of rights and Physical assault by patient	0.068	10.6	0.63
Patients’ bill of rights and Verbal assault by patient	**0.030**	38.9	0.89
Patients’ bill of rights and Physical assault by patients' companion	0.063	10.9	0.70

Significance less than 0.05 (P<0.05) was considered in this study.

## Discussion

One of our constrains was that there was no archival literature regarding the relationship between workplace violence and patients’ bill of rights; therefore, our findings just compared the results of workplace violence with other published articles.

It is worth mentioning that Iran is mostly resided by the Muslims and exact data about sexual harassment or cultural violence are not available. Due to such limitations, among all types of violence, just verbal and physical assaults against nurses and nurses' assistants are discussed in this study and the rest are left for future research. 

Our results showed that 83.1%, 22.1%, 88.3%, and 31.2% of nurses and nurse assistants had experienced verbal or physical violence by patients/their attendants. It seems that our findings are consistent with that of other authors.^24-26^ However, Jacobson^27^ reported the rates of 97% and 74% for physical and verbal assaults, respectively. Such different estimations of verbal and physical assaults could be justified through various measuring instruments used by the researchers. Although, Nulan et al. and Lawoko et al.^28-29^ reported that male nurses were more vulnerable than female ones, others^30-32^ observed higher physical violence against female nurses than the males. The difference could be justified by observing the fact that women are much more respectful than men, and also cultural atmosphere does not allow men to attack the females, at least in Iran. So, it can be said that physical violence against women in Iran is much lower than that of other countries; on the other hand, the female nurses encounter verbal assaults. In national^33-34^ and present studies, it was seen that most of the violent events were originated from patients' attendants. However, other studies^35-36^ showed that patients used to offend more than their visitors/ attendants, and the difference could be due to intensive security processes in western countries for patients’ visitors.

The patients/attendants weapon's threat was moderate in this study compared to other means; however, the prevalence rate of 30.5% for such a mean rate has been reported in Iran.^33^ It was observed that nurses did not like to report violence officially, except when they were hurt. Hence, most reports were on physical violence because some nurses believed that verbal assault is a part of their job.^26,34,37^ Although most of the nurses thought that reporting aggressive events was not useful, some of them who reported physical assaults did not officially pursuit their complaints due to time constrains and other reasons.^38-40^ In the present study, it was found that violators were more of non-traumatic patients' attendants, non-trauma patients, trauma patients' visitors, and trauma patients, respectively, which shows a good agreement with that of Salimi et al.'s.^34^ Regarding the gender of violators and time of violence, it was observed that most assaults were committed by the males. Based on the time, evening and night shifts at no vacation days were reported as the most frequent times of violent events which are completely consistent with Salami's findings.^34^

Lack of enough employees as snursing staff, insufficient security guards, doctors' delay in visiting the patients, insufficient facilities of the hospitals, and improper patient's care were the most common reasons of violence against nurses, respectively, which are in agreement with those of Rahmani et al's.^41,42^ Almost all the offended nursing staff urged the violators to keep calm, but some violators did not care, so some nurses self-defends were inevitable. Such behaviors are in line with the hypothesis that most of the nurses have accepted such workplace violence as a part of their job. 

## Conclusion

Our results showed that verbal and physical assaults of patients’ attendants were much more prevalent than that of the patients themselves. It has been observed that men were at higher risk than women for physical violence, and women were at greater risk than men for verbal assaults.^34^ Hence, complete removal of workplace violence seems superficial and it is suggested to identify the violation factors, and then safe workplaces must be provided in all hospitals. It is suggested to employ security staff for each ward, and also to issue visiting card for patients' relatives/visitors. Furthermore, employing enough nursing staff, improving their salary, and providing the opportunity to attend educational workshops on aggression management are recommended. Further, in educational curriculum of nursing, some papers should be included on violence management. However, the development of a specific office to record, manage and follow up violence against the staff and to promote patients’ bill of rights is suggested. 
